# Solvent Replacement‐Driven Ionic Liquid Thermoelectric Gel for Self‐Powered Morse Code Communication Assisted by Machine Learning

**DOI:** 10.1002/advs.202509400

**Published:** 2025-07-13

**Authors:** Lingshuang Kong, Fengrui Zhao, Jing Li, Fanlun Meng, Wenlong Xu

**Affiliations:** ^1^ Department of Materials Science and Engineering Ludong University Yantai 264025 China

**Keywords:** gel, Ionic liquid, machine learning, thermoelectric conversion, solvent replacement

## Abstract

The development of ionic liquid gels (IL gels) with both high thermoelectric performance and mechanical flexibility is essential for advancing low‐grade heat energy harvesting in next‐generation flexible electronics and self‐powered systems. Herein, a poly(methacrylic acid) (PMAA)‐based IL gel is reported, fabricated via a solvent replacement strategy. By tailoring the synergistic coordination between Fe^2+^/Fe^3+^ redox couples and carboxyl (─COOH) groups in the polymer network, the gel functions as a thermogalvanic electrolyte, and its voltage generation is driven by temperature‐dependent redox reactions of Fe^2+^/Fe^3+^. The resulting gel demonstrates excellent thermoelectric stability over a broad temperature range, achieving a high ionic conductivity (σ) of 13.45 S·m^−1^ and the Seebeck coefficient (S_i_) of −4.67 mV·K^−1^. In situ Raman spectroscopy and low‐field solid‐state nuclear magnetic resonance (NMR) analysis reveal the directional migration behavior of Fe^2+^/Fe^3+^ ions under a thermal gradient and their dynamic coupling with polymer chain motion. Furthermore, a self‐powered Morse‐code communication system is developed using a machine learning (ML)‐assisted framework. A logistic regression model achieved 100% accuracy on an independent test set, indicating a strict monotonic mapping between voltage signals and encoded characters. This work provides new insights into the molecular design and thermoelectric regulation mechanisms of flexible thermoelectric gel, paving the way for their practical application in wearable self‐powered communication devices.

## Graphical abstract

A solvent replacement method is employed to transform PMAA hydrogel into a PMAA IL gel. The assembled thermoelectric device demonstrated stable voltage output and temperature sensitivity across a wide operating temperature range. Additionally, a self‐powered communication system integrating the PMAA IL gel with a logistic regression model is developed, enabling real‐time, energy‐free communication for individuals with speech impairments.

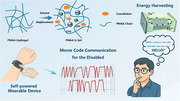

## 1 Introduction

As the global energy crisis intensifies and the push for carbon neutrality accelerates, the development of efficient and scalable thermoelectric conversion technologies has emerged as a research frontier in the field of energy materials. Thermoelectric materials can directly and efficiently convert low‐grade heat sources (such as industrial waste heat and human body heat) into electrical energy, showing broad application potential in wearable electronics, self‐powered sensors, and flexible energy harvesting systems.^[^ ^1^, ^2^ ^]^ Although traditional inorganic thermoelectric materials (e.g., Bi_2_Te_3_, PbTe) exhibit excellent thermoelectric performance at room temperature, their high thermal conductivity, intrinsic brittleness, and complex high‐temperature processing severely limit their adoption in flexible and lightweight devices.^[^ ^3^ ^]^ Therefore, the development of novel material systems that simultaneously offer good mechanical flexibility, environmental stability, and high thermoelectric conversion efficiency has become key to advancing the practical application of thermoelectric technologies.

In recent years, flexible organic‐ionic liquid composite gels based on ion migration mechanisms have attracted widespread attention due to their low thermal conductivity, tunable ionic structures, and excellent adaptability to mechanical deformation.^[^ ^4^, ^5^, ^6^ ^]^ These materials typically rely on a thermoelectric effect driven by the synergistic action of ionic thermodiffusion and redox reactions, offering a conversion pathway fundamentally different from that of traditional electronic thermoelectric mechanisms. However, current ionic thermoelectric gels commonly suffer from uneven distribution of ion migration channels, complex interfacial polarization behavior, and poor thermal response stability.^[^ ^7^ ^]^ In particular, under the coupling of multiscale dynamic strain and temperature gradients, their thermoelectric performance tends to fluctuate and deteriorate significantly. This not only limits the energy conversion efficiency of gel materials in wide temperature ranges and high‐frequency operational environments, but also highlights the lack of effective strategies for regulating interfacial charge transport mechanisms.

To address the aforementioned bottlenecks, current research has focused on the synergistic optimization of polymer network structure design and ionic liquid functional integration, aiming to regulate ion diffusion dynamics and interfacial electronic state distribution.^[^ ^8^, ^9^ ^]^ Among various polymers, PMAA with its programmable distribution of ─COOH groups and abundant hydrogen bonding sites, can form a highly crosslinked 3D network. This structure offers significant advantages in stabilizing ionic liquids and facilitating interfacial synergy.^[^ ^10^ ^]^ Additionally, the redox couple Fe^2+^/Fe^3+^, known for its high electrochemical activity, can enhance potential output under thermal gradients via reversible electron transfer, and induce increased ionic σ under combined electrical and thermal stimuli.^[^ ^11^ ^]^ However, conventional preparation methods (such as solution blending or in situ polymerization) often lead to microstructural heterogeneities such as phase separation and ionic clustering, resulting in a “performance coupling paradox” between mechanical strength and conductivity. This trade‐off hinders the simultaneous optimization of thermoelectric efficiency and structural stability.^[^ ^12^ ^]^ For instance, the composite gel reported by Lu *et al.* achieved a high ionic σ of 13.58 S·m^−1^, but its low tensile strength made it unsuitable for applications involving complex deformation;^[^ ^13^ ^]^ similarly, the Fe^2+^/Fe^3+^ thermoelectric gel developed by Liu *et al.* exhibited more than 30% fluctuation in its S_i_ within the 30–60 °C range, indicating that its thermoelectric response was highly temperature‐dependent and unstable.^[^ ^14^ ^]^ These findings highlight the urgent need for systematic investigation and breakthroughs in the compatibility regulation between ionic liquids and polymer networks, dynamic migration behaviors of redox couples, and associated energy dissipation mechanisms at the molecular level.

This work proposes a novel strategy for constructing PMAA ionic liquid gels (PMAA IL gels) based on a solvent‐replacement‐induced infiltration mechanism. For the first time, the highly polar ionic liquid [Bmim][FeCl_4_] is introduced into a preformed PMAA hydrogel network, enabling uniform migration and stable incorporation of the ionic liquid within the polymer's 3D porous channels. Through synergistic coordination between Fe^2+^/Fe^3+^ redox couples and the ─COOH groups of PMAA, the strategy effectively enhances interfacial energy dissipation and stabilizes ion conduction pathways. This approach not only significantly improves the ionic mobility and thermoelectric conversion performance of the gel, but also mitigates mechanical fatigue and conductivity degradation under repeated deformation. Experimental results demonstrate that the gel exhibits a stable ionic σ of up to 13.45 S·m^−1^ and a negative S_i_ (−4.67 mV·K^−1^) within the temperature range of 0–70 °C. In situ Raman spectroscopy and low‐field solid‐state NMR further reveal the directional migration behavior of Fe^2+^/Fe^3+^ under a temperature gradient, elucidating the microscopic physical mechanism underlying the thermoelectric effect within the gel. It is important to clarify that the present system operates via a thermogalvanic mechanism, where the redox couple Fe^2+^/Fe^3+^ undergoes oxidation and reduction at the hot and cold electrodes, respectively. This is distinct from ionic thermoelectric systems governed by the Soret effect, which involve asymmetric ionic diffusion without electron transfer. In our system, voltage generation primarily arises from redox potential gradients, thus categorizing it as a thermogalvanic cell. Building upon these findings, we integrated the IL gels into a self‐powered flexible device for Morse code communication. Using a logistic regression model, we established a monotonic mapping between thermoelectric voltage signals and encoded letters, achieving 100% classification accuracy on an independent test set. This study not only provides a theoretical foundation for material and interface design in high‐performance flexible thermoelectric gels, but also lays the groundwork for the development of next‐generation flexible self‐powered electronic devices.

## 2 Results and Discussion

### 2.1 Preparation and Characterization of PMAA IL Gels

In this work, the ionic liquid [Bmim][FeCl_4_] was successfully synthesized through simple vigorous stirring to meet the requirements for solvent replacement. To analyze its structural characteristics, the synthesized product was characterized using Fourier‐Transform Infrared Spectroscopy (FTIR). As shown in Figure S1a (Supporting Information), in the FTIR spectrum of [Bmim]Cl, the peak ≈1166 cm^−1^ corresponds to the stretching vibration absorption of the C─N bond within the imidazole ring, the peak near 1464 cm^−1^ is attributed to the main chain vibration of the imidazole ring, and the peak at 1564 cm^−1^ is assigned to the stretching vibration of the C═N bond in the imidazole ring.^[^ ^15^ ^]^ It is noteworthy that all the aforementioned characteristic peaks in the FTIR spectrum of [Bmim][FeCl_4_] exhibit red shifts, indicating strong interactions between the FeCl_4_^−^ anion and the imidazolium group. In addition, the structure of the synthesized product was further characterized by Raman spectroscopy. As shown in Figure S1b (Supporting Information), the obtained spectrum is consistent with literature reports, in which the peak at 333 cm^−1^ corresponds to the fully symmetric Fe‐Cl stretching vibration absorption of FeCl_4_^−^.^[^ ^16^ ^]^ To further confirm the spectral characteristics of the synthesized product, its UV–vis absorption spectrum was measured. As shown in Figure S1c (Supporting Information), three distinct characteristic absorption peaks appear at 534, 619, and 688 nm, which are well‐known characteristic peaks of FeCl_4_^−^, further confirming the successful synthesis of [Bmim][FeCl_4_].^[^ ^17^ ^]^

**Figure 1:**
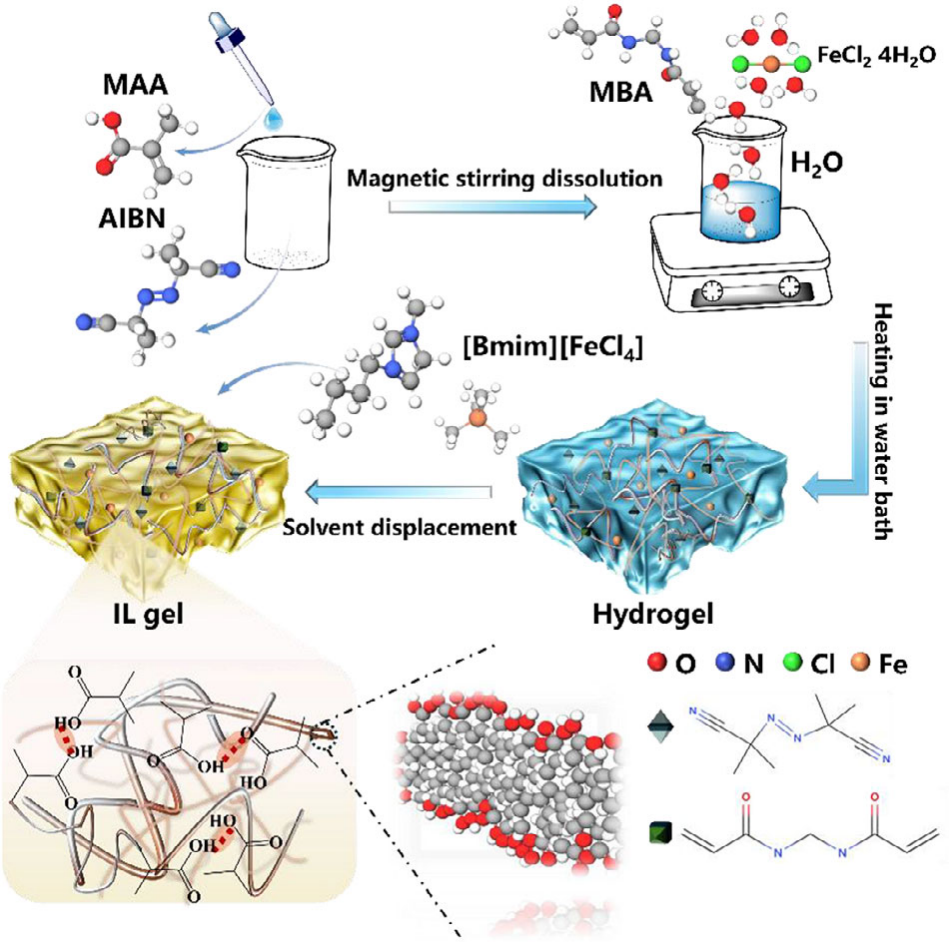
Preparation of PMAA IL gel.

**Figure 2:**
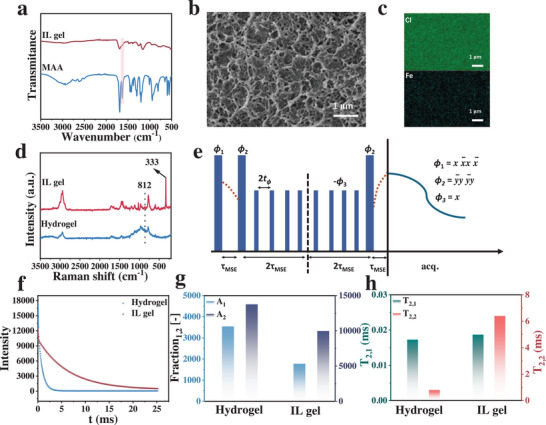
(a) FTIR spectra of MAA and PMAA IL gel; (b) SEM image of PMAA IL gel; (c) EDX mapping of PMAA IL gel; (d) Raman spectra of PMAA IL gel and PMAA hydrogel; (e) MSE‐FID sequence model; (f) FID decay signals of PMAA IL gel and PMAA hydrogel; (g) Relative proportions of relaxation components in PMAA IL gel and PMAA hydrogel; (h) T_2_ values of relaxation components in PMAA IL gel and PMAA hydrogel.

PMAA IL gel was successfully prepared via a solvent replacement strategy, and the synthesis pathway is illustrated in **Figure** **1**. First, methacrylic acid (MAA) undergoes free radical polymerization under thermal initiation to form a 3D network PMAA hydrogel. Subsequently, the water in the PMAA hydrogel is replaced with the ionic liquid [Bmim][FeCl_4_] through a solvent replacement process, thereby yielding the PMAA IL gel. It is worth noting that the introduced ferrous chloride tetrahydrate additive and the ionic liquid [Bmim][FeCl_4_] provide a Fe^2+^/Fe^3+^ redox ion pair to the system, endowing the PMAA IL gel with thermoelectric conversion capability. To verify the polymerization process of MAA, the FTIR spectrum shown in **Figure** **2**a reveals a significant decrease in the intensity of the C═C stretching band of MAA (≈1631 cm^−1^) during the reaction, indicating that MAA has undergone polymerization to form PMAA.^[^ ^18^ ^]^ The Scanning Electron Microscopy (SEM) image shows that the PMAA IL gel exhibits a typical 3D porous network structure, which facilitates the uniform distribution of the ionic liquid and efficient ion migration (Figure 2b). Meanwhile, the SEM‐EDX image further confirms that the Cl and Fe elements from the [Bmim][FeCl_4_] ionic liquid are uniformly distributed throughout the PMAA IL gel (Figure 2c), indicating that the solvent replacement strategy successfully achieved effective incorporation of the ionic liquid. Moreover, as shown in the macroscopic images of PMAA IL gel before and after staining (Figure S2a), the Oil Red O dye exhibits a relatively uniform red coloration throughout the PMAA IL gel. It is noteworthy that Oil Red O is insoluble in water but soluble in the ionic liquid [Bmim][FeCl_4_], and its distinct coloration effectively highlights the degree of staining in the PMAA IL gel. This further confirms the uniform penetration of the ionic liquid within the gel. Similarly, Confocal Laser Scanning Microscopy (CLSM) images of the PMAA IL gel stained with the hydrophobic fluorescent dye 8‐anilino‐1‐naphthalenesulfonic acid ammonium salt (ANS‐NH_4_) (Figure S2b, Supporting Information) also reveal a relatively uniform fluorescence distribution, further confirming the homogeneous infiltration of the ionic liquid. Raman spectral analysis reveals that the peak at 812 cm^−1^ corresponds to the C─O─C stretching vibration of PMAA,^[^ ^19^ ^]^ while the peak at 333 cm^−1^ is attributed to the vibrational absorption of FeCl_4_^−^ (Figure 2d). In the Raman spectrum of the PMAA IL gel, both characteristic absorption peaks are clearly observed, indicating that the ionic liquid [Bmim][FeCl_4_] was successfully introduced during the solvent replacement process. Taken together, these experimental results confirm the successful fabrication of the PMAA IL gel and demonstrate that the gel underwent a thorough and effective solvent replacement.

Low‐field solid‐state NMR primarily investigates information related to intramolecular chemical bonding in the microscopic field.^[^ ^20^ ^]^ Therefore, to elucidate the segmental dynamics of the polymer in situ, the interaction between [Bmim][FeCl_4_] and the polymer network was investigated using low‐field solid‐state NMR spectroscopy. In principle, molecular mobility can be inferred from the simple free induction decay (FID) signal.^[^ ^21^ ^]^ Rapid and slow signal decays indicate the presence of rigid and mobile components, respectively.^[^ ^22^ ^]^ Using the multi‐slice echo‐FID (MSE‐FID) sequence (Figure 2e), a fully refocused FID signal with complete shape can be obtained. By fitting the decay to a multi‐component model, quantitative information about different mobile components within the sample can be extracted. Figure 2f shows the FID signal curves of PMAA hydrogel and PMAA IL gel. The shape fitting of the FID is obtained using the following Equation:

(1) $$f\left(t\right)=A_{1}\exp[-{\left(t/T_{2,1}\right)}^{n_{1}}]+A_{2}\exp\left[-{\left(t/T_{2,2}\right)}^{n_{2}}\right]+C$$

where n_1_ = 2, n_2_ = 1, A_1_ represents the signal intensity of the short relaxation component, A_2_ represents the signal intensity of the long relaxation component; T_2,1_ denotes the T_2_ relaxation time of the short relaxation component, and T_2,2_ denotes the T_2_ relaxation time of the long relaxation component.

Quantitative fitting of the above Equation (1) provides the relative proportions (A) and T_2_ values of the relaxation components for both PMAA IL gel and PMAA hydrogel, as shown in Figure 2g,h. Based on the FID decay signals, both the PMAA IL gel and the PMAA hydrogel exhibit rapidly decaying FID curves. However, the A_1_ and A_2_ of PMAA hydrogel are both larger than those of the PMAA IL gel, indicating that the PMAA hydrogel has a denser pore distribution. In addition, by comparing the T_2_ values, it can be observed that the T_2,2_ value of the PMAA IL gel is larger than that of the PMAA hydrogel, indicating a higher proportion of the long relaxation component in the PMAA IL gel. This suggests that the interaction between [Bmim][FeCl_4_] and the PMAA polymer chains enhances the segmental heterogeneity and mobility of the polymer chains,^[^ ^23^ ^]^ implying a relatively more rigid nature of the PMAA hydrogel and a relatively more mobile structure in the PMAA IL gel.

### 2.2 Thermogalvanic Performance of PMAA IL Gels

**Figure 3:**
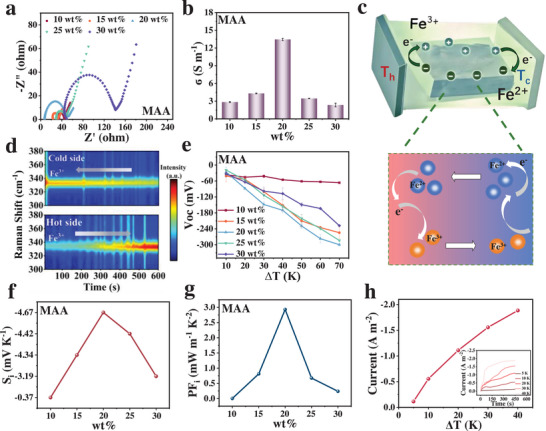
(a) EIS spectrum, (b) σ plot, (c) schematic diagram of the thermoelectric conversion mechanism, (d) in situ Raman spectra, (e) Voc plot, (f) S_i_ plot, (g) PF_i_ plot, and (h) short‐circuit current plot of the PMAA IL gel.

The thermoelectric behavior observed in this study is based on a thermogalvanic process, rather than pure thermodiffusion, with the Fe^2+^/Fe^3+^ redox reaction being the key driving force for voltage generation. The key factors for utilizing PMAA IL gel as the electrolyte material in thermogalvanic cell lie in its thermoelectric conversion performance and σ. First, its σ was thoroughly investigated. As shown in **Figure** **3**a, the impedance variation curves of IL gels with different MAA mass concentrations were obtained via electrochemical impedance spectroscopy (EIS). With increasing MAA mass concentration, the resistance of the IL gel initially decreases and then increases. Using Equation S2 (Supporting Information), the calculated σ exhibits a trend of first increasing and then decreasing, with a maximum value reaching 13.45 S·m^−1^ (Figure 3b). At low MAA concentrations, the formed polymer network is relatively loose with larger internal pores. As the MAA concentration increases, the 3D network structure becomes more complete, providing more effective pathways for ion migration, thereby enhancing σ.^[^ ^24^ ^]^ However, excessive MAA during polymerization results in a denser structure between polymer chains. A higher crosslinking density restricts the segmental mobility of the polymer, limiting ion migration channels and consequently reducing σ.^[^ ^25^ ^]^ The impedance curves of IL gels with different Fe^2+^ mass concentrations are shown in Figure S3a (Supporting Information). As the Fe^2+^ content increases, the concentration of freely mobile ions in the system rises,^[^ ^26^ ^]^ resulting in decreased gel resistance. Consequently, the σ gradually increases (Figure S3b, Supporting Information). The crosslinker directly influences the formation of ion migration channels within the gel. When the crosslinker concentration is too low, the conductive fillers are relatively uniformly distributed in the gel, resulting in lower resistance in the PMAA IL gel and thus higher σ. As the amount of MBA increases, the gel's resistance first decreases and then increases, showing a trend in which σ initially increases and then decreases (Figure S4a,b, Supporting Information). The crosslinker can interact with polymer chains to enhance ion migration efficiency,^[^ ^27^ ^]^ thereby achieving the highest σ. However, when the crosslinker concentration is too high, excessive crosslinking results in an overly dense gel network structure, making it difficult for ions to move within the compact network. This reduces the freedom of ion migration, leading to a decrease in σ.^[^ ^28^ ^]^ In addition, considering the operating temperature range of thermogalvanic cells, the σ of PMAA IL gel was tested from 0 °C to 70 °C. As shown in Figure S5a (Supporting Information), EIS measurements were used to obtain the gel resistance at different temperatures. The corresponding σ values at each temperature were calculated and are displayed in Figure S5b (Supporting Information). It can be observed that σ remains nearly constant or varies only slightly within the 0–70 °C range, indicating that the σ of PMAA IL gel is almost unaffected by environmental temperature. This suggests that the migration of charge carriers in the gel is minimally influenced by temperature and exhibits a low Fermi‐level temperature dependence.^[^ ^29^ ^]^ The σ is close to saturation or has already reached an optimized state, resulting in negligible changes in σ with temperature variation. This indicates that the thermoelectric performance of the material is relatively stable and not easily affected by temperature fluctuations, thus offering long‐term operational stability. Stable σ ensures that the gel maintains high working efficiency even under harsh conditions (such as environments with significant temperature differences), avoiding performance degradation caused by thermal changes. For thermoelectric devices intended for long‐term use‐particularly in scenarios with wide temperature fluctuations‐this material exhibits a clear advantage. Thermogalvanic cells constructed from this gel possess a relatively broad working temperature range, further expanding their potential applications and, to some extent, enhancing their self‐powering capability.

In this study, thermoelectric conversion is achieved through redox reactions of Fe^2+^/Fe^3+^ ion pairs at the interface between the PMAA IL gel and the platinum electrode. Before investigating the thermoelectric conversion performance of the gel, it is essential to understand the redox behavior of the ion pairs within the thermogalvanic cell, as this helps elucidate their role in the thermoelectric conversion process. As shown in Figure 3c and its magnified view, when a temperature gradient is applied across the thermogalvanic cell, the thermoelectric effect leads to enhanced thermal motion of ions at the hot end (T_h_), driving the diffusion of Fe^2+^ (lower oxidation state) toward the hot side, where it is oxidized to Fe^3+^ (higher oxidation state), simultaneously releasing electrons. Correspondingly, a reduction reaction occurs at the cold end (T_c_), where Fe^3+^ is reduced back to Fe^2+^, and these electrons flow through the external circuit to generate current.^[^ ^14^ ^]^ In this process, the redox species consumed at one electrode are replenished by the reverse reaction occurring at the other electrode, maintaining reaction continuity and enabling sustained self‐powering. Through in situ Raman characterization, the ion concentration distribution within the gel and its dynamic changes with temperature can be directly observed. As shown in Figure 3d, the peak at 333 cm^−1^ corresponds to the vibrational absorption of Fe^3+^. At the cold end, the intensity of this ionic peak gradually decreases over time until it reaches a dynamic equilibrium. In contrast, at the hot end, the peak intensity progressively increases until equilibrium is achieved. This observation indicates that during the thermoelectric conversion process, as described above, Fe^3+^ and Fe^2+^ ions migrate within the PMAA IL gel and undergo redox reactions at the hot and cold ends, respectively, thus enabling thermoelectric conversion. Furthermore, when Fe^3+^ and Fe^2+^ reach a dynamic equilibrium, a stable ion concentration gradient is established, thereby realizing a closed‐loop energy conversion cycle.

The S_i_ is a core parameter used to evaluate the thermoelectric conversion performance of thermoelectric materials, reflecting the potential difference generated by a temperature gradient. S_i_ can be determined by measuring the relationship between the open‐circuit voltage (Voc) and the temperature difference. As shown in Figure 3e, the Voc of PMAA IL gels with different MAA mass concentrations was measured under temperature differences ranging from 10 to 70 K. The cold side was maintained at ≈25 °C, and the hot side temperature was increased up to ≈95 °C, beyond which thermal degradation or moisture loss may occur. By calculating the slope of the Voc‐temperature difference curve, the corresponding S_i_ values were obtained. As illustrated in Figure 3f, with increasing MAA mass concentration, S_i_ initially increases and then decreases. The gel with 20 wt.% MAA exhibits the highest S_i_ value, reaching up to −4.67 mV K^−1^. As the monomer concentration increases, a more complete ion migration pathway is constructed within the polymer network, enhancing ion migration in the gel. However, excessively high monomer concentrations may lead to uneven internal electric field distribution, which can hinder normal thermoelectric conversion and reduce S_i_.^[^ ^30^ ^]^ Notably, thermoelectric materials constructed using the Fe^2+^/Fe^3+^ redox couple belong to n‐type thermoelectric materials, and thus their S_i_ values are negative. This is mainly because, in the thermoelectric conversion process, electron carriers serve as the primary charge transport mechanism, leading to a negative voltage output.^[^ ^31^ ^]^ Specifically, during the oxidation of Fe^2+^ to Fe^3+^, free electrons are released, increasing the electron concentration in the system and conferring n‐type conductivity to the material. Electron hopping between Fe^2+^ and Fe^3+^ (small polaron conduction) enhances electron mobility, thereby strengthening the n‐type behavior.^[^ ^32^ ^]^ Additionally, Fe^2+^/Fe^3+^ may coordinate with the ─COOH groups of PMAA to form metal‐organic framework (MOF‐like) structures,^[^ ^33^ ^]^ which enhances the delocalization of electrons in the Fe‐O coordination bonds. The power factor (PF_i_), calculated using Equation S3 (Supporting Information), is shown in Figure 3g and follows a trend similar to that of S_i_. Moreover, the cyclic voltammetry (CV) curves of gels with different MAA mass concentrations also indicate the extent of the redox reactions, indirectly reflecting the trend of S_i_ values (Figure S6, Supporting Information).

The short‐circuit current refers to the current that flows through a thermoelectric material when there is a temperature difference across its two ends and no external load is applied to the circuit.^[^ ^34^ ^]^ The generation of this current is related to the Seebeck effect of the material, and its magnitude reflects the material's responsiveness to temperature changes. In practical applications, thermogalvanic cells are often subjected to various degrees of temperature differences, especially under dynamic thermal conditions or changing environmental factors. Therefore, testing the short‐circuit current can also reveal the thermogalvanic cell's adaptability to temperature gradients. Based on this, the short‐circuit current of the PMAA IL gel was tested under temperature differences ranging from 5 to 40 K. As shown in Figure 3h, the short‐circuit current increases with increasing temperature difference. A higher current indicates a better thermoelectric conversion capability of the gel.

Fe^2+^ directly participates in the redox reactions during the thermoelectric conversion process, thus having a direct influence on the thermoelectric performance. The Voc of PMAA IL gels with varying Fe^2+^ mass concentrations was measured under temperature differences ranging from 10 to 70 K (Figure S7a, Supporting Information). By calculation, the S_i_ was obtained, which shows a trend of initially increasing and then decreasing with the rising Fe^2+^ concentration (Figure S7b, Supporting Information). Correspondingly, the degree of redox reactions also first intensifies and then weakens, as shown in Figure S7c (Supporting Information). As the Fe^2+^ concentration increases, the concentration of charge carriers (i.e., Fe^2+^ ions) in the material also rises, which facilitates electron migration, promotes the development of an electric potential difference, and thus improves the S_i_. However, when the concentration of Fe^2+^ ions becomes too high, excessive Fe^2+^ may lead to aggregation, which hinders the effective migration of ions within the material.^[^ ^35^ ^]^ Although the charge carrier concentration remains high under these conditions, ion aggregation and excessive migration suppress S_i_, resulting in decreased thermoelectric performance.

In addition, the cross‐linker MBA can regulate the cross‐linking density of the polymer network in the gel, thereby affecting its thermoelectric conversion performance. The Voc and S_i_ of PMAA IL gels with different MBA mass concentrations were tested and calculated (Figure S8a,b, Supporting Information). As the MBA concentration increases, S_i_ first increases and then decreases, corresponding to the redox reaction intensity, which also initially enhances and then weakens (Figure S8c, Supporting Information). When the cross‐linker concentration is relatively low, the gel network structure is loose and gradually becomes more complete, allowing ions to migrate more freely under a temperature gradient. At this point, the gel exhibits a stronger thermoelectric effect, resulting in an increased S_i_. However, when the MBA concentration is too high, the gel network becomes overly dense. Excessive cross‐linking narrows the ion transport channels, thereby reducing the S_i_.

In summary, the PMAA IL gel exhibits relatively stable conductivity and thermoelectric conversion performance, enabling efficient thermoelectric self‐powering over a wide temperature range. To contextualize the thermogalvanic performance of our system, we compared the σ and S_i_ with other reported thermogalvanic electrolytes. As shown in Table S1 (Supporting Information), our PMAA IL gel exhibits a high σ of 13.45 S·m^−1^ and a negative S_i_ of −4.67 mV·K^−1^, comparable to or exceeding those of previously reported Fe^2+^/Fe^3+^ systems. Moreover, the gel form offers improved mechanical flexibility and integration potential for wearable electronics compared to liquid‐based systems.

### 2.3 Mechanical Properties of PMAA IL Gels

The prepared PMAA IL gel exhibits excellent flexibility, which contributes to enhancing the safety of thermogalvanic cells by reducing issues such as electrolyte rupture or leakage caused by mechanical stress. Moreover, the highly flexible gel can better accommodate the volume changes that occur during the self‐powering process of thermogalvanic cells, thereby improving their durability.

**Figure 4:**
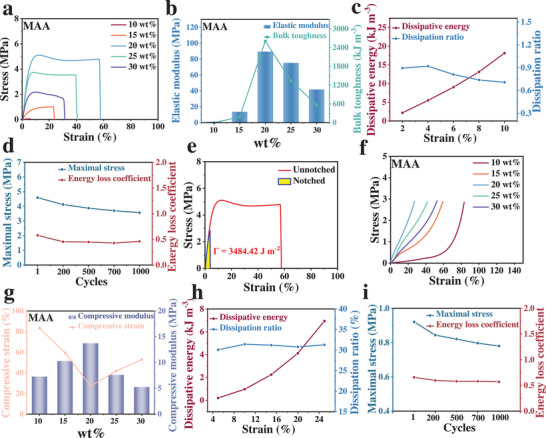
Mechanical properties of PMAA IL gels with different MAA mass concentrations: (a) Uniaxial tensile stress‐strain curves, (b) elastic modulus and toughness, (c) progressive tensile dissipated energy and dissipation ratio and (d) maximum stress and energy dissipation coefficient plot during cyclic tensile testing; (e) Single‐edge notch test; (f) Uniaxial compression curves, (g) compressive strain and compressive modulus, (h) progressive compression dissipated energy and dissipation ratio, and (i) maximum stress and energy dissipation coefficient during cyclic compression testing.

The mechanical properties of PMAA IL gels can be regulated by adjusting the mass ratio of MAA. The uniaxial tensile curves of PMAA IL gels with different MAA concentrations are shown in **Figure** **4**a. As the MAA mass concentration increases, the tensile strength of the PMAA IL gels first increases and then decreases. This trend is mainly attributed to the balance between the crosslinked network structure of the gel, intermolecular interactions, and mechanical performance. With an increasing concentration of MAA, more monomers participate in the polymerization reaction, resulting in a higher crosslinking density within the gel, which leads to a denser network structure.^[^ ^36^ ^]^ In a moderately crosslinked network, molecular chains are able to slide to some extent, which helps disperse external forces and enhance the gel's extensibility. Meanwhile, the ─COOH in the MAA molecules can form hydrogen bonds or electrostatic interactions with the polar groups of the cations in [Bmim][FeCl_4_], creating a stable network structure that further enhances the tensile strength of the gel. However, when the MAA monomer concentration becomes too high, the crosslinking density of the gel increases excessively, resulting in an overly rigid internal network. This leads to a decrease in flexibility and extensibility of the gel, making it more prone to fracture under tension, thereby reducing its tensile strength.^[^ ^37^ ^]^ Alternatively, an excessively high crosslinking density limits the mobility of polymer chains, preventing effective dispersion of tensile stress, which also results in a decline in overall tensile strength.^[^ ^38^ ^]^ It is noteworthy that most of the stress‐strain curves exhibit a period of smooth linearity, indicating strong tensile resistance. This behavior is mainly related to the stretching and disentanglement of molecular chains within the gel network. When molecular chains are stretched to a certain extent under stress, they may gradually disentangle or slide from a disordered state, leading to a redistribution of intermolecular interactions within the gel network.^[^ ^39^ ^]^ During the disentanglement process, PMAA IL gels exhibit lower rigidity, resulting in minimal changes in external stress while strain continues to increase. Further analysis of the stress‐strain curves enables the calculation of the elastic modulus and toughness of the PMAA IL gels. As shown in Figure 4b, both parameters follow a trend of first increasing and then decreasing with increasing MAA mass concentration.

Based on the above observations, the PMAA IL gel with an MAA concentration of 20 wt.% was selected for further analysis of its dynamic and reversible energy dissipation behavior. Progressive tensile and 1000‐cycle tensile tests were conducted. In the progressive tensile curves, the presence of residual strain during the unloading process indicates irreversible energy dissipation behaviors, which reflect molecular slippage, network rearrangement, or the breaking of crosslinking points.^[^ ^40^ ^]^ As shown in Figure S9a (Supporting Information), the residual strain increases with strain, indicating that the molecular chains of the gel gradually transition from an initially disordered state to a more aligned and sliding configuration. During this process, the dissipated energy increases from 2.18 to 18.15 kJ m^−3^, as shown in Figure 4c. In cyclic tensile testing, hysteresis loops are typically observed. The area of each hysteresis loop represents the energy loss (i.e., dissipated energy) during the loading and unloading process.^[^ ^41^ ^]^ A larger loop area suggests more pronounced inelastic deformation and energy dissipation within the gel. As shown in Figure S9b (Supporting Information) and Figure 4d, after multiple cycles, both the stress and hysteresis loop area decrease and then stabilize, reflecting the evolution of the energy dissipation mechanism with the number of cycles and indicating that a steady‐state dissipation mechanism is eventually established. To further evaluate the fracture toughness and crack propagation behavior of the PMAA IL gel, a single‐edge notch test was performed (Figure 4e). External force was applied at the notch, and the gel absorbed deformation energy before the onset of crack propagation. The gel then fractured along the direction of the notch. In this process, the PMAA IL gel achieved a fatigue threshold of 3484.42 J m^−2^. These results demonstrate that the PMAA IL gel possesses excellent tensile properties.

Compression performance tests were also conducted on PMAA IL gels with varying MAA mass concentrations. Through uniaxial compression experiments, the effect of MAA concentration on the compressive strength of the gels was analyzed (Figure 4f). The results show that as the MAA concentration increases, the compressive strength of the gel initially increases and then decreases. Specifically, at the same stress level, the compressive strain first decreases and then increases, while the compressive modulus exhibits a trend of first increasing and then decreasing (Figure 4g). This variation trend is consistent with the tensile behavior and is also closely related to the crosslinking density. For the PMAA IL gel with 20 wt.% MAA concentration, progressive compression, and cyclic compression tests were carried out to investigate its plastic deformation, elastic recovery behavior, and energy dissipation capacity. In the progressive compression experiment, as shown in Figure S9c (Supporting Information), with increasing compression strain, the gel underwent elastic deformation followed by a yielding stage, yet still maintained a relatively stable dissipation radio (Figure 4h). In the cyclic compression test, the PMAA IL gel maintained a relatively stable energy dissipation coefficient over 1000 compression cycles, indicating excellent long‐term stability and durability (Figure S9d, Supporting Information; Figure 4i).

Fe^2+^ ions in ferrous chloride tetrahydrate can coordinate with functional groups (e.g., ─COOH) in the PMAA IL gel molecules, thereby influencing the crosslinking of the polymer chains. To investigate this effect, uniaxial tensile and compression tests were performed on PMAA IL gels with varying Fe^2+^ mass concentrations. In the uniaxial tensile tests, as the Fe^2+^ concentration increased from 0 to 7 wt.%, the tensile strength of the gels gradually decreased (Figure S10a, Supporting Information). Meanwhile, the toughness also diminished accordingly (Figure S10b, Supporting Information). This behavior is possibly due to the introduction of Fe^2+^ ions leading to ionic aggregation within the gel matrix, which affects the structural uniformity of the material. As a result, certain regions may become overly compact or rigid, while others remain relatively loose. During stretching, some regions may experience stress concentration, thereby reducing the overall tensile strength of the gel.^[^ ^42^ ^]^ In contrast, gels without Fe^2+^ exhibit more homogeneous structures and can better distribute external forces. In the uniaxial compression tests, as shown in Figure S10c,d (Supporting Information), the compressive strength of the gels also gradually decreased with increasing Fe^2+^ concentration.

The concentration of the crosslinker has a significant impact on the mechanical properties of hydrogels. Appropriate crosslinking can enhance the mechanical strength and stability of the gel, thereby improving its tensile, compressive, and shear resistance. Uniaxial tensile and compression tests were conducted to evaluate the influence of the crosslinker MBA on the mechanical properties of PMAA IL gels. As shown in Figure S11a,b (Supporting Information), with increasing MBA mass concentration, the tensile strength of the gel initially increases and then decreases. Similarly, both the elastic modulus and toughness exhibit a trend of rising first and then falling. When the crosslinker concentration is low, increasing its concentration enhances the crosslinking density of the gel. A greater number of crosslinking points strengthens the interactions between polymer chains, leading to a more stable structure and improved tensile strength.^[^ ^43^ ^]^ However, as the crosslinker concentration continues to rise, excessive crosslinking can occur, resulting in a rigid network. This rigidity compromises the material's extensibility and flexibility, ultimately causing a decline in mechanical performance at higher crosslinker concentrations.^[^ ^44^ ^]^ For the same reasons, the compressive strength of PMAA IL gels also decreases as the MBA mass concentration increases, as illustrated in Figure S11c,d (Supporting Information).

During the preparation of hydrogels, the ratio between different components has a significant impact on the final properties of the gel. To achieve optimal mechanical and thermoelectric performance, it is essential to adjust the proportion of each component according to specific application requirements. Meanwhile, to broaden the application scope of thermogalvanic cells, the freezing resistance of the PMAA IL gel represents another crucial factor under investigation, as detailed in Figure S12 (Supporting Information).

### 2.4 Performance of PMAA IL Gels as Thermogalvanic Cell

A thermogalvanic cell can be constructed by combining the PMAA IL gel electrolyte with two platinum electrodes. The practical performance of the device can be evaluated by testing its thermoelectric output under different temperature differences, as well as its stability under mechanical strain and repeated thermal cycling.

**Figure 5:**
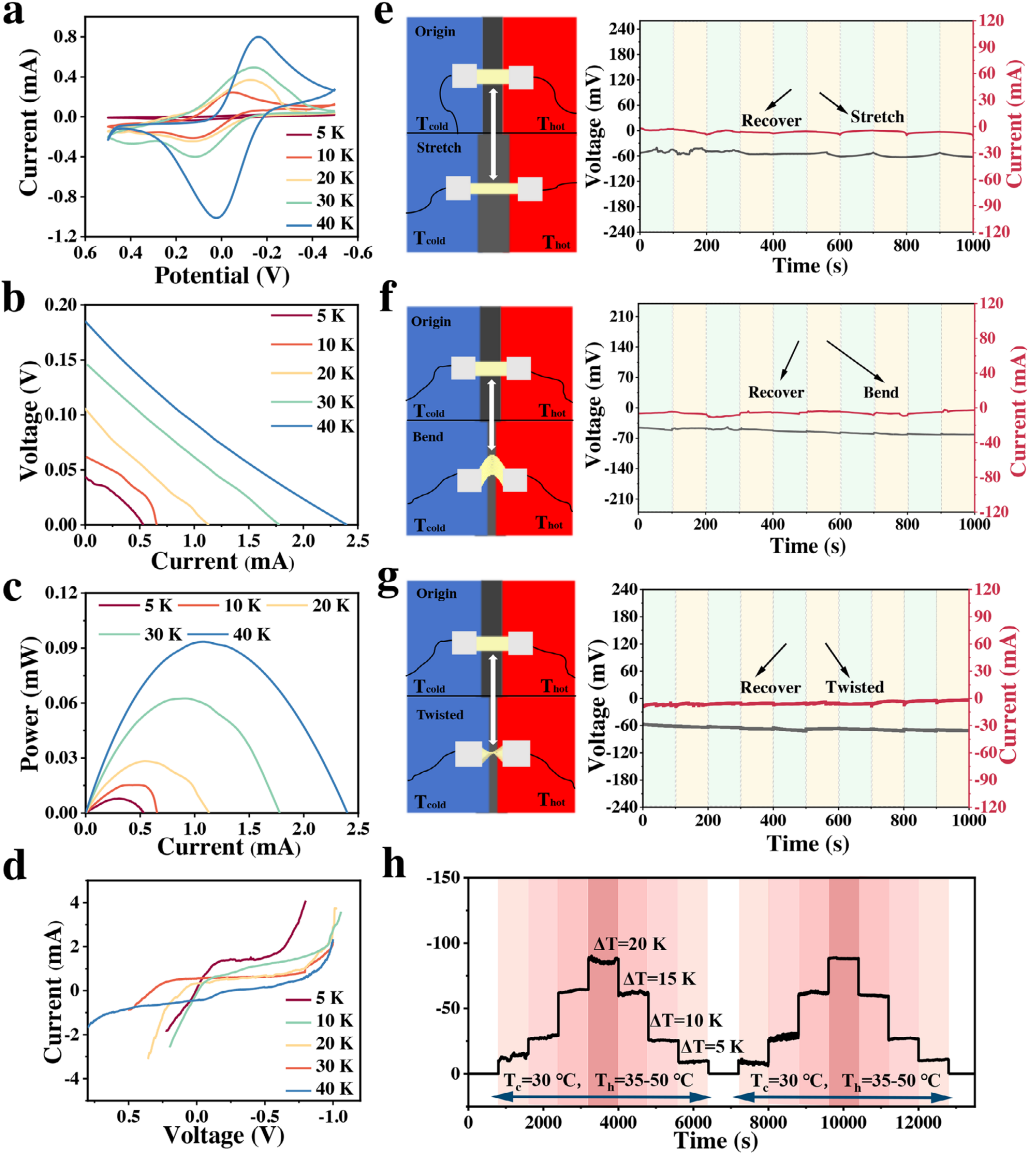
Thermoelectric performance of PMAA IL gel. (a) CV curves, (b) current–voltage (I–V) curves, (c) current‐power curves, and (d) decomposition voltage curves under different temperature differences; Output current/voltage stability under different mechanical strain conditions: (e) repeated 40% tensile strain, (f) repeated 60° bending, and (g) repeated 180° twisting; (h) Output voltage stability under different thermal cycling conditions.

First, the thermoelectric output performance of the thermogalvanic cell was measured under different temperature differences. As shown in **Figure** **5**a, under temperature differences ranging from 5 to 40 K, the intensity and efficiency of the redox reaction in the PMAA IL gel gradually increases. The reaction intensity is generally proportional to the electromotive force. At larger temperature differences, the driving electromotive force is higher, which promotes charge separation and electron flow in the cell, thereby increasing the output power. This indicates that the thermogalvanic cell can achieve good self‐powered performance across a relatively wide temperature range in practical applications. The current response of the thermogalvanic cell under different voltages was measured using linear sweep voltammetry (LSV), and the output power was further calculated. As the voltage increased linearly, the corresponding current response of the cell was recorded. Based on the current‐voltage relationship, the voltammogram is obtained (Figure 5b). At different voltage points on the LSV curve, the output power was calculated using the Equation P = IV (where P represents power, I represents current, and V represents voltage). As shown in Figure 5c, with increasing temperature difference, the maximum output power provided by the thermogalvanic cell also increases. The decomposition voltage refers to the maximum voltage that the thermogalvanic cell can provide under different temperature differences when no external load is applied.^[^ ^45^ ^]^ The redox reactions in the thermogalvanic cell driven the decomposition voltage, which varied with temperature difference. As shown in Figure 5d, the decomposition voltage increases as the temperature difference rises. This is because the temperature difference is the main driving force for the thermoelectric effect: with increasing temperature gradients, ion migration and potential differences are enhanced, resulting in higher voltage output.

Second, the stability of the thermogalvanic cell under mechanical strain and repeated thermal cycling was investigated. Testing the voltage/current stability of the thermogalvanic cell under mechanical deformation is a key performance indicator for evaluating its applicability in real‐world scenarios, especially in the fields of flexible electronics and wearable devices. Mechanical strain (particularly stretching or bending) may affect the thermoelectric performance of the cell. Under a fixed temperature difference of 10 K, the thermogalvanic cell was subjected to repeated cycles of 40% tensile strain. As shown in Figure 5e, the cell maintains relatively stable voltage and current output throughout the stretching cycles, indicating that the thermoelectric performance is highly tolerant to tensile deformation. Similarly, when the cell was subjected to repeated 60° bending cycles, it still maintains relatively stable output voltage and current values (Figure 5f). Additionally, repeated 180° twisting shows comparable results (Figure 5g). These findings demonstrate that the thermogalvanic cell exhibits excellent mechanical strain stability and can maintain reliable performance in real‐world applications.

The thermal cycling stability of the thermogalvanic cell was also evaluated to assess its reliability and long‐term durability under practical usage. Thermal cycling may lead to performance degradation of thermoelectric materials, especially under repeated heating and cooling conditions. To assess this, the output voltage of the cell was measured over time while varying the temperature difference (0–20 K). As shown in Figure 5h, during the time interval of 0–6800 s, the cold and hot electrodes are both maintained at 30 °C (T_c_) for the first 800 s. Then, the temperature of the hot electrode is increased in 5 °C increments every 800 s up to 50 °C. Subsequently, the hot electrode is cooled back to 30 °C to restore a 0 K temperature difference, and the cycle is repeated. Within the full duration of 0–13 600 s, the output voltage increases from 0 to −89 mV, then decreases back to 0 mV, and the cycle is repeated. This demonstrates that the thermogalvanic cell exhibits rapid thermal responsiveness to dynamic temperature differences. Moreover, the output voltage remains relatively stable in each thermal cycle, indicating excellent thermal cycle stability and recovery capability. This suggests strong potential for long‐term use, especially in applications where thermogalvanic cells are exposed to continuously changing environmental temperatures.

### 2.5 ML‐Assisted Self‐Powered Communication System Based on PMAA IL Gel

To demonstrate the practical applicability of the developed PMAA IL gel, we further constructed a ML‐assisted, thermoelectric self‐powered communication interface, in which the PMAA IL gel acts as both the energy‐harvesting unit and signal generator. This system enables real‐time, energy‐autonomous communication for individuals with speech disabilities and offers a promising solution in the field of self‐powered sensing and human‐machine interaction.

**Figure 6:**
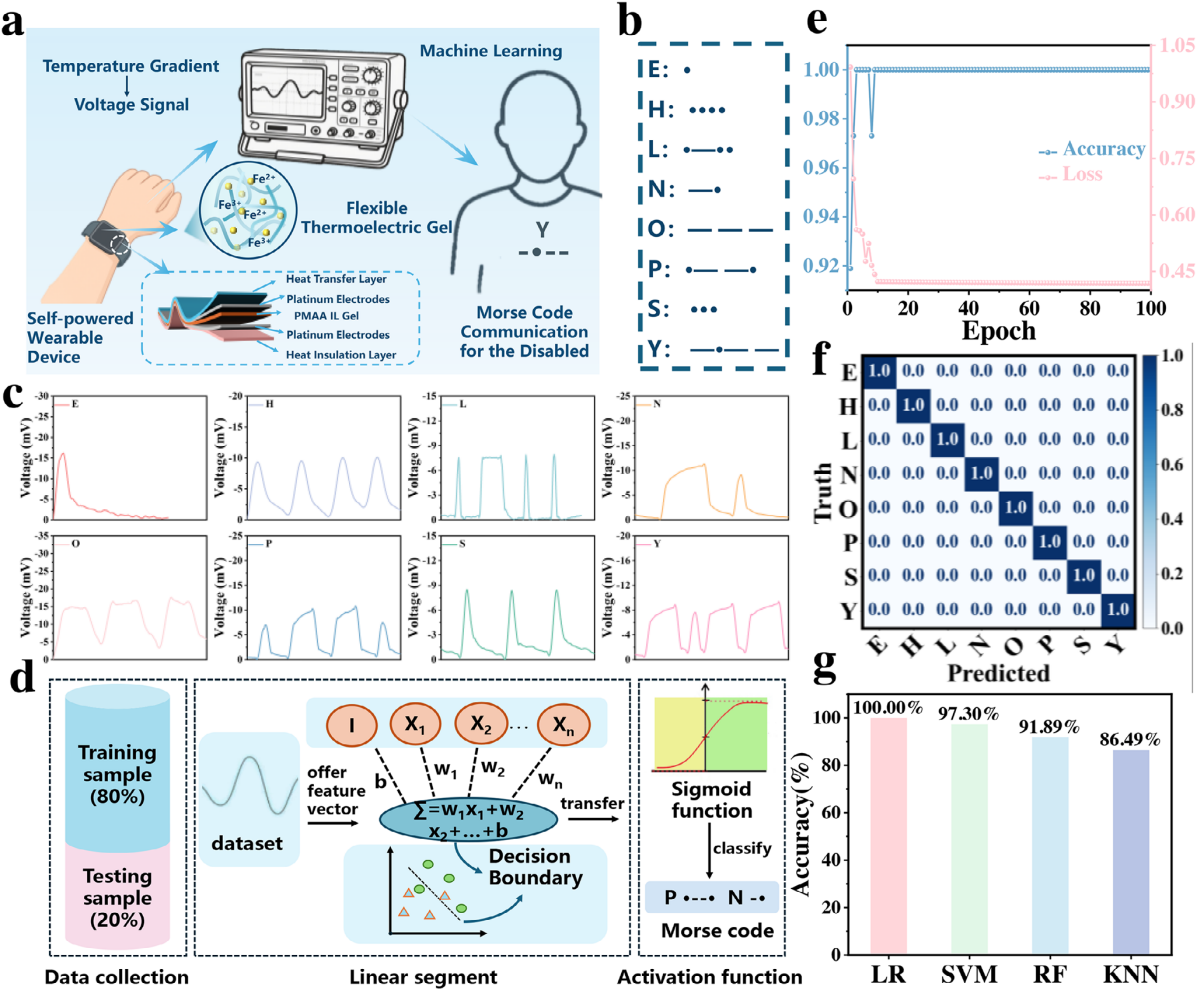
(a) Schematic illustration of the thermoelectric self‐powered accessible communication system; (b) Eight letters in Morse code selected for training; (c) Voltage waveforms corresponding to the eight Morse code letters; (d) LR model framework for the accessible communication system; (e) Accuracy and loss values over 100 training iterations for the LR model; (f) Confusion matrix of the LR model; (g) Comparison of classification accuracies among four ML models.

As illustrated in **Figure** **6**a, a wearable gel‐based wristband was fabricated, capable of generating thermovoltage waveforms when worn on the arm. Upon physical contact from the other hand, the thermal gradient across the gel induces a thermoelectric voltage signal, whose duration and amplitude are determined by the contact time and thermal difference. The system was trained to recognize eight frequently used letters in Morse code (E, H, L, N, O, P, S, Y) (Figure 6b), whose corresponding waveform patterns were used as input features for the ML model. These letters were selected based on their frequent occurrence in commonly used emergency or assistive communication words (e.g., “HELP,” “YES,” and “NO”),^[^ ^46^, ^47^ ^]^ as well as the distinguishability of their thermoelectric signal patterns in preliminary experiments. Due to the redox‐driven nature of the PMAA IL gel, longer contact times result in more extensive Fe^2+^/Fe^3+^ redox activity, generating longer amplitude voltage outputs that can be clearly differentiated (Figure 6c; Figure S13 and Video S1, Supporting Information). To classify the thermoelectric signals, a logistic regression (LR) model was developed. The system architecture consists of three main stages: data collection, linear segment, and activation function (Figure 6d). The dataset was split into 80% training and 20% testing subsets. During training, input feature vectors are projected into a higher‐dimensional space via a linear transformation using learned weights. These linear combinations are then passed through a Sigmoid activation function, converting the outputs into probabilistic values between 0 and 1. A predefined threshold is used to convert the probability into a discrete classification decision, which correlates with a specific letter. This process mirrors Morse‐code logic and enables fully self‐powered, symbolic communication.

After training and evaluation, the LR model achieved 100% accuracy within just eight iterations, maintaining small classification loss throughout the training process (Figure 6e), highlighting the consistency and high fidelity of the voltage waveforms. A confusion matrix further confirmed that all eight Morse‐coded voltage signals were correctly classified without error (Figure 6f). To benchmark the performance of the LR model, we also applied three commonly used ML algorithms: Support Vector Machine (SVM), Random Forest (RF), and K‐Nearest Neighbors (KNN). However, their confusion matrices revealed significantly lower classification accuracies compared to LR (Figure S14, Supporting Information). The performance ranking across models was LR > SVM > RF > KNN, indicating that the quasi‐linear voltage response generated by the redox process in PMAA IL gels aligns optimally with the linear decision boundary of the LR algorithm. Among the models, SVM achieved the second‐highest accuracy due to its effectiveness in maximizing class margins in low‐dimensional, well‐structured feature space. RF outperformed KNN due to its higher robustness to feature noise and its reduced risk of overfitting in small‐sample settings. To further illustrate the nature of the classification problem, we visualized the feature space using Principal Component Analysis (PCA) and plotted decision boundaries for the four models (Figure S15, Supporting Information). These plots confirm the margin‐separable structure of the data and explain the relative performance across classifiers.

In summary, we successfully developed a self‐powered, ML–enhanced communication system using PMAA IL gels and logistic regression modeling. This system enables real‐time Morse code communication without any external power supply, providing a practical, wearable solution for assistive communication in speech‐impaired populations. The synergy between thermoelectric chemistry and AI‐driven signal recognition demonstrates a compelling direction for the next generation of intelligent, energy‐autonomous soft electronics.

## 3 Conclusion

In summary, we have developed a novel PMAA IL gel system via a solvent replacement strategy, enabling the effective incorporation of the highly polar ionic liquid [Bmim][FeCl_4_] into a 3D polymer network. The resulting gel exhibits outstanding thermoelectric performance, with a high ionic σ of 13.45 S·m^−1^ and a stable negative S_i_ of −4.67 mV·K^−1^ across a broad temperature range (0–70 °C). Mechanistic investigations using in situ Raman spectroscopy and low‐field solid‐state NMR have revealed a temperature‐driven, directional migration of Fe^2+^/Fe^3+^ redox couples and their synergistic coordination with PMAA ─COOH groups, which together enhance interfacial ion transport and energy dissipation. Moreover, by integrating the gel into a flexible thermoelectric device, we demonstrated a self‐powered Morse code communication system assisted by machine learning. The trained logistic regression model achieved perfect decoding accuracy, confirming the feasibility of real‐time, barrier‐free communication based on thermoelectric signal modulation. This work not only provides a new design strategy for high‐performance, flexible thermoelectric gels, but also opens new avenues for wearable electronics and intelligent human‐machine interfaces powered by low‐grade thermal energy.

## Conflict of Interest

The authors declare no conflict of interest.

## Supporting information



Supporting Information

Supplemental Video 1

## Data Availability

The data that support the findings of this study are available from the corresponding author upon reasonable request.
