# Supplementary material for: Solvent Replacement‐Driven Ionic Liquid Thermoelectric Gel for Self‐Powered Morse Code Communication Assisted by Machine Learning
Source: Adv Sci (Weinh). 2025 Jul 13;12(38):e09400. doi: 10.1002/advs.202509400 (PMC12520506; doi:10.1002/advs.202509400)
Supplement: Supplementary file 1 — Supporting Information [file ADVS-12-e09400-s001.docx]

**Supporting Information**

**Solvent Replacement-Driven Ionic Liquid Thermoelectric Gel for Self-Powered Morse Code Communication Assisted by Machine Learning**

*Lingshuang Kong, Fengrui Zhao, Jing Li, Fanlun Meng, Wenlong Xu**

Department of Materials Science and Engineering, Ludong University, Yantai 264025, China

*Corresponding authors:

E-mail: [xuwenlong@ldu.edu.cn](mailto:xuwenlong@ldu.edu.cn)

**Experimental Section**

**Materials.** Azobisisobutyronitrile (AIBN, 99%) and N, N'-methylenebisacrylamide (MBA, 99%) were purchased from J&K Scientific Co., Ltd. Methyl methacrylate (MAA, 98%), 1-butyl-3-methylimidazolium chloride ([Bmim]Cl, 97%), ANS-NH_4_, anhydrous ferric chloride (FeCl_3_, 99%), and ferrous chloride tetrahydrate (98%) were obtained from Shanghai Macklin Biochemical Co., Ltd. Oil Red O dye was purchased from Fuzhou Feijing Biotechnology Co., Ltd.

**Preparation of [Bmim][FeCl_4_] Ionic Liquid.** [Bmim]Cl and FeCl_3_ were mixed at a molar ratio of 1:1 and vigorously stirred at room temperature for 8 h to obtain [Bmim][FeCl_4_].

**Preparation of [Bmim][FeCl_4_] IL Gel.** First, 0.5 wt% AIBN was mixed with 20 wt% MAA and stirred until the AIBN was completely dissolved. Then, 77.5 wt% ultrapure water and 1 wt% MBA were added and stirred until a clear and transparent solution was obtained. After adding 1 wt% ferrous chloride tetrahydrate and allowing it to fully dissolve, the mixture was placed in a 60  ℃ water bath and heated for 3 h to obtain PMAA hydrogel. The hydrogel was then immersed in [Bmim][FeCl_4_] for a 9 h solvent replacement process to obtain the PMAA IL gel. PMAA IL gels with other concentrations were prepared following the same procedure, but using different amounts of MAA (10 wt%, 15 wt%, 25 wt%, 30 wt%), ferrous chloride tetrahydrate (0 wt%, 3 wt%, 5 wt%, 7 wt%), or MBA (0.1 wt%, 0.5 wt%, 3 wt%, 5 wt%).

**Thermoelectric Performance Testing of PMAA IL Gels.** EIS, chronopotentiometry, LSV, CV, and current-time (I-t measurements were carried out using an electrochemical workstation (CHI660E, Chenhua Instruments, Shanghai, China). The heating and cooling panels used to generate temperature gradients were self-assembled. The resistance was obtained from the first intersection point of the EIS plot with the horizontal axis. The ionic conductivity (σ) was calculated using the following Equation:

$\text{σ=L/(R×S)}$ (2)

L represents the distance between two adjacent electrodes, R denotes the resistance of the sample, and S refers to the cross-sectional area of the hydrogel sample.

The PF_i_ was calculated using the following Equation:

$\text{PF}_{\text{i}}\text{=}\text{σ×}\text{S}_{\text{i}}^{\text{2}}$ (3)

σ represents electrical conductivity and S_i_ represents Seebeck coefficient.

**Mechanical Performance Testing of PMAA IL Gel.** The mechanical strength of the PMAA IL gel was measured using a universal testing machine (Nss, Shenzhen Laboratory Equipment Co., Ltd., China). Both tensile and compressive tests were conducted in a uniaxial mode. The tensile test was carried out at a speed of 50 mm/min, and the compression test at 5 mm/min. The tensile test specimens were dumbbell-shaped, with a length of 20 mm, width of 10 mm, and thickness of 2 mm. The compression specimens were cylindrical, with a diameter of 22 mm and a thickness of 10 mm. The tensile toughness and elastic modulus of the PMAA IL gel were calculated respectively from the area under the tensile stress-strain curve (via line integration) and from the slope of the curve. To further evaluate the energy dissipation properties of the gel, progressive and cyclic loading tests were performed. During cyclic tests, the energy dissipation ratio was calculated as the ratio of the area enclosed by the hysteresis loop to the area under the loading curve. In the single-edge notch test, a 1 mm notch was introduced on the edge of the specimen perpendicular to the stretching direction. The stretching speed was set at 50 mm/min. The fracture energy (Γ) was calculated using the following Equation:

$\text{Γ=6Wc/√}\lambda_{\text{c}}$ (4)

λ_c_ is the fracture stretch ratio of the notched specimen (λ_c_ = ε_c_ + 1), where ε_c_ is the fracture strain of the notched specimen, c is the notch length, and W is the overall toughness calculated by integrating the stress-strain curve of an unnotched specimen with the same dimensions stretched to ε_c_.

**Antifreeze Performance Testing of PMAA IL Gel.** The antifreeze performance of PMAA IL gels was evaluated using a universal testing machine (Nss, Shenzhen Laboratory Equipment Co., Ltd., China). Uniaxial tensile and compressive tests were conducted on PMAA IL gels with different degrees of solvent replacement: irreplaced, replaced for 5 h, and replaced for 9 h. The tensile toughness and elastic modulus were calculated based on the area under the stress-strain curves (line integral) and the slope of the curves, respectively.

**Characterization.** The chemical structures of [Bmim]Cl, [Bmim][FeCl_4_], MAA, and PMAA IL gels were investigated using a Nicolet™ iS50 Fourier Transform Infrared (FTIR) spectrometer (Thermo Fisher Scientific, USA). The synthesis of [Bmim][FeCl_4_] was verified by Evolution One Plus UV-Vis spectrophotometer (Thermo Fisher Scientific, USA). The degree of solvent replacement in PMAA IL gels was analyzed by confocal laser scanning microscopy (CLSM, Olympus FV3000) after staining with ANS-NH_4_. The internal microstructure of the PMAA IL gels was observed using a Hitachi SU8010 field emission scanning electron microscope (Hitachi, Japan). Elemental mapping of the gels was conducted via EDX analysis using a ZEISS Sigma 360 scanning electron microscope (Zeiss, Germany). Raman spectra of [Bmim][FeCl_4_], PMAA IL gels, and PMAA hydrogels were recorded with a LabRAM HR800 confocal Raman spectrometer (HORIBA, Japan). Ion concentration variations between the hot and cold sides of PMAA IL gels were further monitored using an inVia confocal Raman microscope (Renishaw, UK). The relaxation times and the relative fractions of mobile components in PMAA hydrogels and IL gels were characterized by a low-field solid-state NMR spectrometer (VTMR20010V-I) from Suzhou Niumag Corporation, China. The crystallization temperatures of PMAA IL gels under different degrees of solvent replacement were determined using Differential Scanning Calorimetry (DSC) (NETZSCH, Germany). The antifreeze performance of the gels was evaluated by Dynamic Mechanical Analysis (DMA242E), also from NETZSCH, Germany.

**Figure S1.** (a) FTIR spectra of [Bmim]Cl and [Bmim][FeCl_4_]; (b) Raman spectrum of [Bmim][FeCl_4_]; (c) UV-Vis absorption spectrum of [Bmim][FeCl_4_].

**Figure S2.** (a) Macroscopic images before and after staining of the PMAA IL gel; (b) CLSM image of the PMAA IL gel.

**Figure S3.** (a) EIS spectra and (b) σ plots of PMAA IL gels with different Fe^2+^ mass concentrations.

**Figure S4.** (a) EIS spectra and (b) σ plots of PMAA IL gels with different MBA mass concentrations.

**Figure S5.** (a) EIS spectra and (b) σ plots of PMAA IL gels at different temperatures.


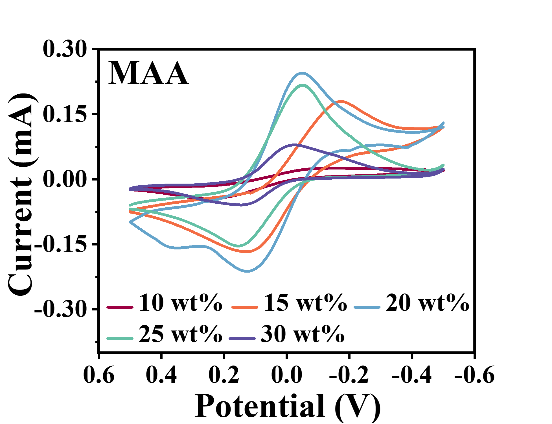


**Figure S6.** CV curves of PMAA IL gels with different MAA mass concentrations.

**Figure S7.** (a) Voc curves, (b) S_i_ curves, and (c) CV curves of PMAA IL gels with different Fe^2+^ mass concentrations.

**Figure S8.** (a) Voc curves, (b) S_i_ curves, and (c) CV curves of PMAA IL gels with different MBA mass concentrations.

**Figure S9.** (a) Progressive tensile stress-strain curves, (b) Cyclic tensile stress-strain curves, (c) Progressive compression stress-strain curves, (d) Cyclic compression stress-strain curves of PMAA IL gel.

**Figure S10.** (a) Uniaxial tensile stress-strain curves, (b) Elastic modulus and toughness, (c) Uniaxial compression stress-strain curves, (d) Compressive strain and compressive modulus of PMAA IL gel with different Fe^2+^ concentrations.

**Figure S11.** (a) Uniaxial tensile stress-strain curves, (b) Elastic modulus and toughness, (c) Uniaxial compression stress-strain curves, (d) Compressive strain and compressive modulus of PMAA IL gel with different MBA concentrations.

**Antifreeze Performance of PMAA IL Gels**

Testing the antifreeze performance of thermogalvanic cells enables evaluation of their performance stability in low-temperature environments, particularly for practical applications such as wearable devices and field exploration in cold climates. Under low temperatures, the stability, efficiency, and operational performance of thermogalvanic cells directly affect their long-term reliability.

The degree of solvent replacement in PMAA IL gels is crucial to the antifreeze performance of thermogalvanic cells. Incomplete replacement may lead to crystallization of the gel electrolyte under low-temperature conditions, resulting in changes to the internal structure of the gel. This not only affects the mechanical properties of the gel but also severely impairs ion migration within the gel and the redox reactions at the gel-electrode interface, thereby hindering the conversion of thermal energy into electrical energy. Based on this, we first evaluated the crystallization temperatures of gel samples with three different solvent replacement times using Differential Scanning Calorimetry (DSC) analysis. As shown in **Figure S12a**, as the replacement time increases from 0 h to 5 h, and finally to 9 h, the crystallization temperature of the gel samples progressively decreases. Within the range of -50  ℃ to 0 ℃, no crystallization peak appears for sample replaced for 9 h. The position and intensity of the freezing crystallization peak at low temperatures can indicate the degree of ice crystal formation in the gel. A lower freezing point suggests better antifreeze performance. Furthermore, this result indirectly indicates that a 9-hour replacement time is sufficient to completely convert PMAA hydrogel into PMAA IL gel.

Furthermore, Dynamic Mechanical Analysis (DMA) was used to analyze the changes in mechanical properties of the three samples mentioned above under low-temperature conditions (**Figure S12b**). The storage modulus (E') reflects the elastic performance of the material, indicating the rigidity and elasticity of the gel at low temperatures^[1]^. As the temperature decreases, the E' values of samples replaced for 0 h and 5 h increase significantly, suggesting that the gels become stiffer during cooling. This may be due to structural changes caused by water crystallization, indicating poor antifreeze performance. The loss modulus (E'') reflects the viscous dissipation characteristics of the gel^[2]^. Changes in E'' under low temperatures reveal energy dissipation caused by restricted molecular motion. Sample replaced for 9 h exhibits a relatively low loss modulus, indicating better flexibility and superior antifreeze capability. At -50  ℃, from the perspective of mechanical performance, the uniaxial tensile and compressive strength curves of samples replaced for 0 h and 5 h show significant increases compared to the samples replaced for 9 h (**Figures S12c and S12e**). In contrast, the stress-strain behavior of sample replaced for 9 h remains nearly consistent with its room-temperature results (**Figures 4a and 4f**), indicating that water crystallization has occurred in samples replaced for 0 h and 5 h, while sample replaced for 9 h maintains its structure and exhibits excellent antifreeze properties. Further calculations were conducted to obtain the elastic modulus, toughness, compressive strain, and compressive modulus for the three samples, as shown in **Figures S12d and S12f**. The parameters for samples replaced for 0 h and 5 h increase significantly, while those for sample replaced for 9 h remains essentially unchanged. These results demonstrate that thermogalvanic cells constructed using sample replaced for 9 h can maintain good stability even in low-temperature environments.

Antifreeze performance testing of the thermogalvanic cell under low-temperature conditions allows for the assessment of its practical application potential in harsh environments. Good antifreeze properties help enhance the lifespan and stability of thermogalvanic cells in cold environments, which is of great significance for applications that must withstand extreme temperatures, such as wearable devices, field exploration, and aerospace.

**Figure S12.** Antifreeze performance of PMAA IL gel replaced for different time intervals. (a) DSC curves; (b) DMA curves; (c) Uniaxial tensile curves at -50 ℃; (d) Elastic modulus and toughness at -50 ℃; (e) Uniaxial compression curves at -50  ℃; (f) Compressive strain and compressive modulus at -50  ℃.


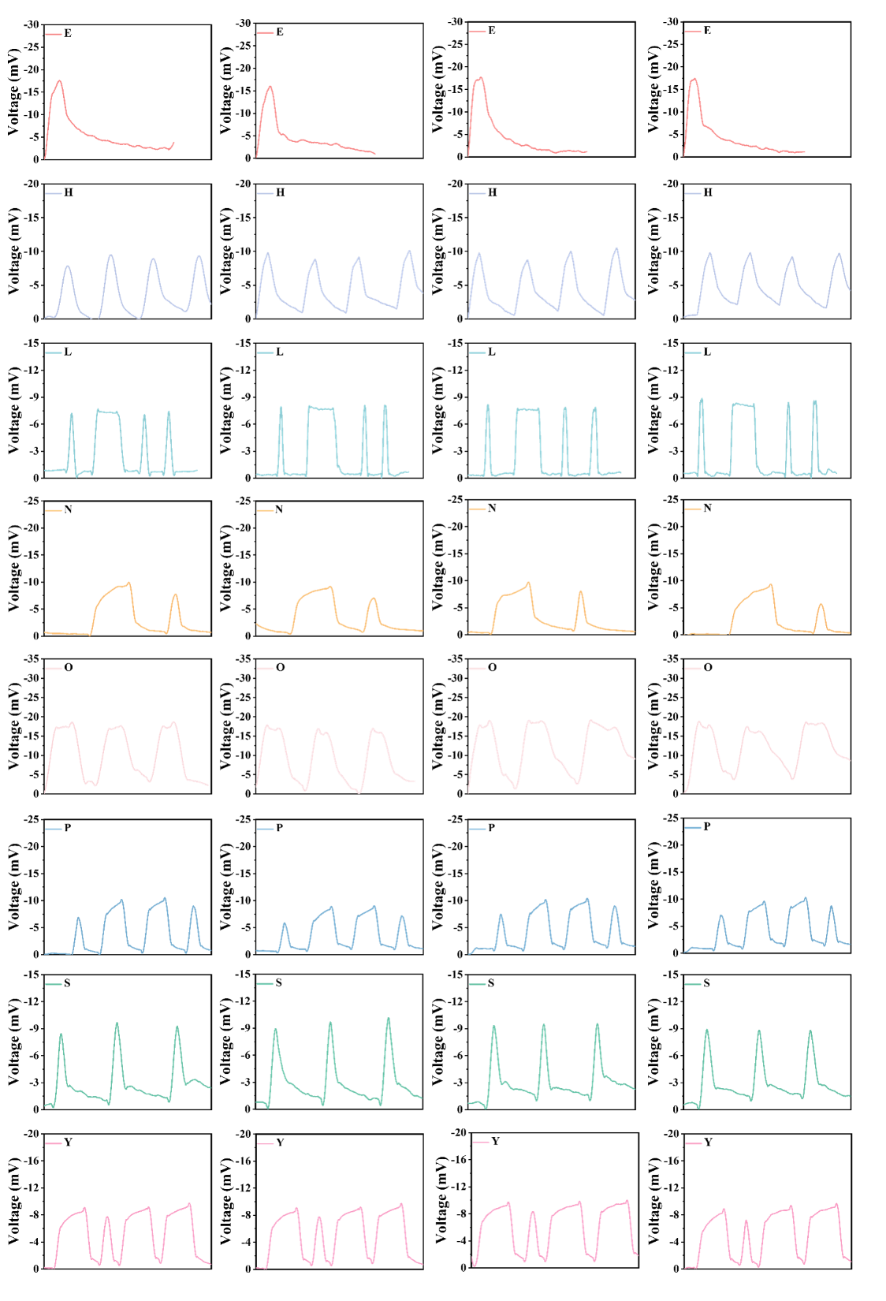


**Figure S13.** Partial voltage waveform training set.

**Figure S14.** Confusion matrices of multiple ML models: (a) SVM model, (b) RF model, and (c) KNN model.

**Figure S15.** Decision boundaries of the four classifiers (LR, SVM, RF, KNN) based on 2D projections.

**Table S1.** Comparison of Representative Thermogalvanic Systems (n-Type).

| **System** | **σ (S·m^-1^)** | **S_i_ (mV·K^-1^)** | ***Ref.*** |
| --- | --- | --- | --- |
| PMAA IL Gel (Fe^2+^/Fe^3+^) | 13.45 | -4.67 | This Work |
| Solvent-tuned Fe^2+^/Fe^3+^ Hydrogel |  | -2.49 | ^[3]^ |
| PVA Hydrogel (Fe^2+^/Fe^3+^) | ~8 | -2.5 | ^[4]^ |
| PVDF/HFP-EMIM:TFSI iTE Gel |  | -4 | ^[5]^ |
| Fe^2+^/Fe^3+^ Aqueous Solution | ~0.5-1 | ~-1 | ^[6]^ |
| BC/PAA/GdmCl Gel (Fe(ClO_4_)_2/3_) |  | -2.8 | ^[7]^ |
| CMCs-PAM DN Gel (Fe^2+^/Fe^3+^) |  | -1.2 | ^[8]^ |
| BC Hydrogel (Fe^2+^/Fe^3+^) |  | -4.5 | ^[9]^ |
| Chitosan Gel (Fe^2+^/Fe^3+^) |  | -7.24 | ^[10]^ |

**Supporting Video**

Video S1：SOS Morse code voltage waveform signal acquisition.

**Machine Learning Program**

import matplotlib

matplotlib.use('TkAgg')

import warnings

from sklearn.preprocessing import LabelEncoder

import cv2

import os

import numpy as np

import matplotlib.pyplot as plt

from sklearn.model_selection import train_test_split

from sklearn.preprocessing import StandardScaler

from sklearn.metrics import accuracy_score, classification_report, confusion_matrix, ConfusionMatrixDisplay

from sklearn.ensemble import RandomForestClassifier

from sklearn.svm import SVC

from sklearn.neighbors import KNeighborsClassifier

from sklearn.linear_model import LogisticRegression

font_properties = {

'fontsize': 42,

'fontweight': 'bold',

'family': 'Times New Roman'

}

default_size = 30

line_width = 5

font_family = 'Times New Roman'

x_label = "Predicted"

y_label = "True Label"

classifiers = {

"SVM": SVC(kernel='rbf', C=1, gamma='scale', random_state=42),

"Random Forest": RandomForestClassifier(n_estimators=100, max_depth=1, random_state=42),

"KNN": KNeighborsClassifier(n_neighbors=5, weights='distance'),

"Logistic Regression": LogisticRegression(max_iter=1000, C=0.1, random_state=42)

}

warnings.filterwarnings("ignore", category=UserWarning)

def load_image_dataset(root_dir, target_size=(100, 100)):

"""Load image dataset, store images by class and return label encoder"""

images, labels = [], []

for class_name in os.listdir(root_dir):

class_dir = os.path.join(root_dir, class_name)

if not os.path.isdir(class_dir):

continue

for filename in os.listdir(class_dir):

file_path = os.path.join(class_dir, filename)

img = cv2.imread(file_path)

if img is not None:

img = cv2.resize(img, target_size)

img = cv2.cvtColor(img, cv2.COLOR_BGR2RGB) # Convert to RGB

img = img.astype(np.float32) / 255.0 # Normalize

images.append(img)

labels.append(class_name)

else:

print(f"Warning: Could not read file {file_path}, skipping")

label_encoder = LabelEncoder()

encoded_labels = label_encoder.fit_transform(labels)

return np.array(images), encoded_labels, label_encoder

def compare_classifiers(images, labels, label_encoder, test_size=0.2, random_state=42):

"""Compare performance of different classifiers"""

X = images.reshape((images.shape[0], -1))

y = labels

X_train, X_test, y_train, y_test = train_test_split(

X, y, test_size=test_size, random_state=random_state, stratify=y)

scaler = StandardScaler()

X_train = scaler.fit_transform(X_train)

X_test = scaler.transform(X_test)

results = {}

class_names = label_encoder.classes_

for name, clf in classifiers.items():

print(f"\nTraining {name}...")

clf.fit(X_train, y_train)

y_pred = clf.predict(X_test)

acc = accuracy_score(y_test, y_pred)

cm = confusion_matrix(y_test, y_pred, normalize='true')

results[name] = {"accuracy": acc, "confusion_matrix": cm, "classifier": clf}

print(f"{name} accuracy: {acc:.4f}")

fig, ax = plt.subplots(figsize=(10, 8))

disp = ConfusionMatrixDisplay(confusion_matrix=cm, display_labels=class_names)

disp.plot(cmap=plt.cm.Blues, values_format=".1f", ax=ax, colorbar=False)

ax.set_xlabel(x_label, **font_properties)

ax.set_ylabel(y_label, **font_properties)

ax.set_xticklabels(class_names, rotation=45, **font_properties)

ax.set_yticklabels(class_names, **font_properties)

for text in disp.ax_.texts:

text.set_fontfamily(font_family)

text.set_fontweight("bold")

text.set_fontsize(default_size)

cbar = fig.colorbar(disp.im_, ax=ax, fraction=0.046, pad=0.02)

cbar.ax.tick_params(labelsize=12)

for label in cbar.ax.get_yticklabels():

label.set_fontfamily(font_family)

label.set_fontweight("bold")

label.set_fontsize(default_size)

for spine in ax.spines.values():

spine.set_linewidth(line_width)

plt.tight_layout()

plt.show()

return results

if __name__ == "__main__":

ROOT_DIR = "./dataset" # Dataset root directory

TARGET_SIZE = (64, 64)

# Load data

images, labels, label_encoder = load_image_dataset(ROOT_DIR, TARGET_SIZE)

print(f"Image data range: min={np.min(images)}, max={np.max(images)}")

# Compare classifiers

results = compare_classifiers(images, labels, label_encoder)

# Print model performance

print("\nModel performance comparison:")

print("{:<20} {:<10}".format('Model', 'Test Accuracy'))

print("-" * 30)

for name, data in results.items():

print("{:<20} {:.4f}".format(name, data['accuracy']))

# Find best model

best_model_name = max(results, key=lambda x: results[x]['accuracy'])

best_model = results[best_model_name]['classifier']

print(f"\nBest model: {best_model_name} (Accuracy: {results[best_model_name]['accuracy']:.4f})")

**Reference**

[1] Z. Feng, M. Xie, J. Lai, Z. Wang, H. Xia. *Angew. Chem. Int. Ed.* **2025**, 35, e202423712.

[2] J. Cheng, S. Fu, S. Ma, Z. Zhang, C. Ma, G. Zhang. *Adv. Mater.* **2024**, 36, 2411700.

[3] Y. Liu, Q. Zhang, G.O. Odunmbaku, Y. He, Y. Zheng, S. Chen, Y. Zhou, J. Li, M. Li, K. Sun. *J. Mater. Chem. A* **2022**, 10, 19690-19698.

[4] H. Zhang, Y. Du, D. Jing, L. Yang, J. Ji, X. Li. *ACS Appl. Mater. Interfaces* **2023**, 15, 49892-49901.

[5] D. Zhao, A. Martinelli, A. Willfahrt, T. Fischer, D. Bernin, Z.U. Khan, M. Shahi, J. Brill, M.P. Jonsson, S. Fabiano, X. Crispin. *Nat. Commun.* **2019**, 10, 1093.

[6] T. Kim, J.S. Lee, G. Lee, H. Yoon, J. Yoon, T.J. Kang, Y.H. Kim. *Nano Energy* **2017**, 31, 160-167.

[7] Y. Jia, S. Zhang, J. Li, Z. Han, D. Zhang, X. Qu, Z. Wu, H. Wang, S. Chen. *Small* **2024**, 20, 2401427.

[8] Y. Zhou, D. Zhang, S. Zhang, Y. Liu, R. Ma, G. Wallace, J. Chen. *SusMat* **2024**, 4, e225.

[9] Y. Zong, H. Li, X. Li, J. Lou, Q. Ding, Z. Liu, Y. Jiang, W. Han. *Chem. Eng. J.* **2022**, 433, 134550.

[10] J. Hu, J. Wei, J. Li, L. Bai, Y. Liu, Z. Li. *Energy Environ. Sci.* **2024**, 17, 1664-1676.
